# Urinary fatty acid binding protein 3 (uFABP3) is a potential biomarker for peripheral arterial disease

**DOI:** 10.1038/s41598-021-90395-0

**Published:** 2021-05-26

**Authors:** Abdelrahman Zamzam, Muzammil H. Syed, John Harlock, John Eikelboom, Krishna K. Singh, Rawand Abdin, Mohammad Qadura

**Affiliations:** 1 Division of Vascular Surgery, St. Michael’s Hospital, Toronto, ON M5B 1W8 Canada; 2 Department of Surgery, Hamilton General Hospital, Hamilton, Canada; 3 Population Health Research Institute, McMaster University, Hamilton, ON Canada; 4 Department of Medicine, McMaster University, Hamilton, ON L8S 4K1 Canada; 5 Department of Medical Biophysics, Schulich School of Medicine and Dentistry, University of Western Ontario, London, ON N6A 5C1 Canada; 6 Department of Surgery, University of Toronto, Toronto, ON M5S 1A1 Canada; 7 Keenan Research Centre for Biomedical Science, Li Ka Shing Knowledge Institute, St. Michael’s Hospital, Toronto, ON M5B 1W8 Canada

**Keywords:** Biomarkers, Translational research, Diagnostic markers, Peripheral vascular disease

## Abstract

Plasma levels of fatty acid binding protein 3 (pFABP3) are elevated in patients with peripheral artery disease (PAD). Since the kidney filters FABP3 from circulation, we investigated whether urinary fatty acid binding protein 3 (uFABP3) is associated with PAD, and also explored its potential as a diagnostic biomarker for this disease state. A total of 130 patients were recruited from outpatient clinics at St. Michael’s Hospital, comprising of 65 patients with PAD and 65 patients without PAD (non-PAD). Levels of uFABP3 normalized for urine creatinine (uFABP3/uCr) were 1.7-folds higher in patients with PAD [median (IQR) 4.41 (2.79–8.08)] compared with non-PAD controls [median (IQR) 2.49 (1.78–3.12), p-value = 0.001]. Subgroup analysis demonstrated no significant effect of cardiovascular risk factors (age, sex, hypertension, hypercholesteremia, diabetes and smoking) on uFABP3/uCr in both PAD and non-PAD patients. Spearmen correlation studies demonstrated a significant negative correlation between uFABP3/uCr and ABI (ρ = − 0.436; p-value = 0.001). Regression analysis demonstrated that uFABP3/Cr levels were associated with PAD independently of age, sex, hypercholesterolemia, smoking, prior history of coronary arterial disease and Estimated Glomerular Filtration rate (eGFR) [odds ratio: 2.34 (95% confidence interval: 1.47–3.75) p-value < 0.001]. Lastly, receiver operator curve (ROC) analysis demonstrated unadjusted area under the curve (AUC) for uFABP3/Cr of 0.79, which improved to 0.86 after adjusting for eGFR, age, hypercholesteremia, smoking and diabetes. In conclusion, our results demonstrate a strong association between uFABP3/Cr and PAD and suggest the potential of uFABP3/Cr in identifying patients with PAD.

## Introduction

Peripheral arterial disease (PAD) is a chronic atherosclerotic condition that affects over 200 million people globally^1–3^. Despite its prevalence, a large proportion of patients with PAD receive delayed treatment, and consequently, face a high risk of lower-extremity amputations and mortality^4–7^.

Currently, the ankle brachial index (ABI)—the ratio of the brachial artery blood pressure to the ankle blood pressure—is the only validated screening tool for PAD^8^. However, the ABI has limitations; it is often erroneously interpreted in primary care, and can be unreliable in patients with diabetes^9–11^.

We have previously demonstrated an association between elevated plasma levels of fatty acid binding protein 3 (pFABP3) and PAD^12^. FABP3 is a small intracellular protein that is normally absent from plasma but is released into the circulation following myocardial or skeletal injury^13,14^.

Because the kidneys play an important role in clearing and filtering pFABP3 from circulation^15–17^, we have now investigated whether urine fatty acid binding protein (uFABP3) levels are elevated in patients with PAD and could potentially serve as a diagnostic biomarker for this disease state.

## Materials and methods

### Ethics approval

This study received approval from the research ethics board at St. Michael's Hospital-University of Toronto in Ontario, Canada. Informed verbal and written consent were obtained from all participants, and all methods were carried out in accordance with the relevant guidelines and regulations.

### Patient recruitment

For this pilot study, the first encountered 65 PAD and 65 non-PAD patients presenting to ambulatory clinics at St. Michael’s Hospital between April 2019 and March 2020 were recruited. As previously described^18^, patients with PAD were defined based on an ABI < 0.9, as well as abnormal distal pulses examination with or without claudication. A control group of patients without PAD were also recruited. The non-PAD control group were defined based on an ABI ≥ 0.9, presence of palpable distal pulses, and no clinical history of claudication. TBI (toe brachial index) measurements were performed where ABI values could not be calculated due to non-compressible tibial vessel. Patients with TBI < 0.67 were characterized as having PAD, whereas controls had a TBI of ≥ 0.67.

Patients with a history of chronic kidney disease (stages 3, 4 and 5) or acute limb ischemia, and those with a history within the past 12 months of acute coronary syndrome (ACS), acute congestive heart failure (CHF), uncontrolled arrhythmia or elevated troponins were excluded.

### Baseline measurements and sample collection

We recorded baseline demographics, history of cardiovascular diseases, cardiovascular risk factors, and smoking status^19^. Each patient also underwent lower limb arterial imaging with an ultrasound (US), ABI or toe brachial index (TBI). Patients were defined as having hypercholesterolemia if they had total cholesterol > 5.2 mmol/L, triglyceride > 1.7 mmol/L, or were taking lipid lowering therapy; as having hypertension if they had a systolic blood pressure ≥ 130 mmHg, diastolic pressure ≥ 80 mm Hg, or were taking blood pressure lowering therapy; and as having diabetes if they had a glycosylated hemoglobin A1c ≥ 6.5% or were taking antidiabetic medication. Patients were defined as having renal disease if they had an estimated glomerular filtration rate of less than 60 mL/min/1.73 m^2^. The Estimated Glomerular Filtration rate (eGFR) was calculated for each patient as previously demonstrated by Levey et al.^20^.

Mid-stream urine samples were collected, aliquoted and stored at − 80 °C prior to analysis. Urine samples were thawed slowly on ice prior to analysis.

### Urinary FABP3 multiplex assay

To determine the concentrations of FABP3 levels in urine (uFABP3), samples were examined in duplicate using MILLIPLEX MAP Human Cardiovascular Disease (CVD) Magnetic Bead Panel 1 (EMD-Millipore; Billerica, MA). To minimize any inter-assay variability, all analyses were carried out on the same day. Sample intra-assay and inter-assay CV were < 10%. Prior to any sample analysis, Fluidics Verification and Calibration bead kits (Luminex Corp) were used to calibrate the MagPix analyzer (Luminex Corp; Austin, Texas). At least 50 beads for uFABP3 were acquired using Luminex xPonent software and analyzed using Milliplex Analyst software (v.5.1; EMD-Millipore).

### Measurement of urinary creatinine and normalization of uFABP3

Urine creatinine (uCr) levels were measured at the Core laboratory at St. Michael’s Hospital using the Beckman Coulter AU680 laboratory analyzer (Beckman Coulter; Pasadena, California). The uFABP3 concentration were normalized to uCr to adjust for urinary concentration errors and differences in hydration status, while relying on single-spot urine samples to achieve normalized uFABP3/uCr (μg/g).

### Statistical methods

Data are presented as median and interquartile ranges (IQR). Normality was assessed using the Shapiro–Wilk test. Since the continuous variables were not normally distributed, the Mann–Whitney U or Kruskal–Wallis test was used to evaluate differences between groups. A post-hoc test was used for pairwise comparisons after multiple group testing. Fisher’s exact test or chi-square test was used for categorical variables. In an attempt to compare uFABP3/uCr levels among PAD and non-PAD patients, patients with PAD where stratified based on ABI into mild (0.89–0.75), moderate (0.74–0.50) and severe PAD (< 0.50) groups as per the European Society for Vascular Medicine (ESVM) guidelines on peripheral arterial disease^21^. Logistic regression was used to evaluate the association between a one standard deviation increase in uFABP3/uCr levels and diagnosis of PAD. Z-scores uFABP3/uCr values were used for ease of odds ratio interpretation. Logistic regression models were used to examine the association between uFABP3/uCr and other parameters with the development of PAD. Correlations between ABI (i.e. PAD severity) and uFABP3/uCr were analyzed using Spearman’s correlation. Area under the curve (AUC) from receiver operating characteristic (ROC) analysis was used to determine the ability uFABP3/uCr in distinguishing between patients with PAD and non-PAD patients. Moreover, to better understand the predictive capabilities of uFABP3/uCr, a ROC curve was estimated using probability estimates from a fitted model with PAD regressed on uFABP3/uCr and age, hypercholesteremia, smoking and diabetes. All analyses were carried out at a 5% two-sided significance level and carried out using SPSS software version 23 (SPSS Inc., Chicago, Illinois, USA).

## Results

### Cohort description and levels of uFABP3/uCr in controls and patients with PAD

A total of 130 patients were enrolled in the study. Median age of the overall study participants was 66, and all collected patient demographics were significantly different between the PAD and non-PAD groups except for sex and eGFR (Table 1). Among the 65 patients with PAD, there were 4 patients (6%) with chronic limb threatening ischemia (CLTI) that had an average ABI of 0.54 and median age of 67 years. Relative to patients with PAD, the CLTI patients did not have any significant difference in measured demographic variables or cardiovascular risk factors. The uFABP3/uCr levels in the PAD group [median (IQR) 4.41 (2.79–8.08)] were 1.7 folds higher than levels of the non-PAD group [median (IQR) 2.49 (1.78–3.12)]. In additional analysis, we investigated the levels of the non-normalized uFABP3 in the PAD group. Our results demonstrated that the non-normalized uFABP3 in the PAD group [median (IQR) 3.48 (2.51–5.45), μg/g] were also significantly higher than in non-PAD controls [median (IQR) 1.89 (0.93–3.40), μg/g].

**Table 1:** Patient demographics and clinical characteristics.

Variable	Non-PAD (n = 65)	PAD (n = 65)	p-value^α^
**Median (IQR)**^**†**^			
ABI	1.06 (1.00–1.13)	0.52 (0.45–0.69)	0.001
Age in years	56 (43–68)	72 (65–80)	0.001
eGFR	88 (72.5–99.5)	86 (69.0–92.0)	0.293
**Frequency, n (%)**^‡^			
Sex-male	36 (55)	45 (69)	0.103
Hypertension	30 (46)	50 (77)	0.001
Hypercholesteremia	23 (35)	49 (75)	0.001
Diabetes	10 (15)	33 (51)	0.001
Smoking	39 (60)	54 (83)	0.004
History of CAD	7 (11)	28 (44)	0.001
History of stroke	1 (2)	10 (15)	0.005

Medians and interquartile ranges (IQR) were calculated for continuous variables.

Frequencies and percentages were calculated for categorical variables; all numbers were rounded up with zero decimal place.

All p-values were rounded to three decimal places.

*ABI* ankle brachial index, *eGFR* estimated glomerular filtration rate.

^α^The significance of the difference between PAD and non-PAD groups.

^†^Differences between groups were compared using Mann–Whitney test.

^‡^Differences between groups were compared using chi-square test.

### Correlation between uFABP3/uCr and PAD

**Figure 1:**
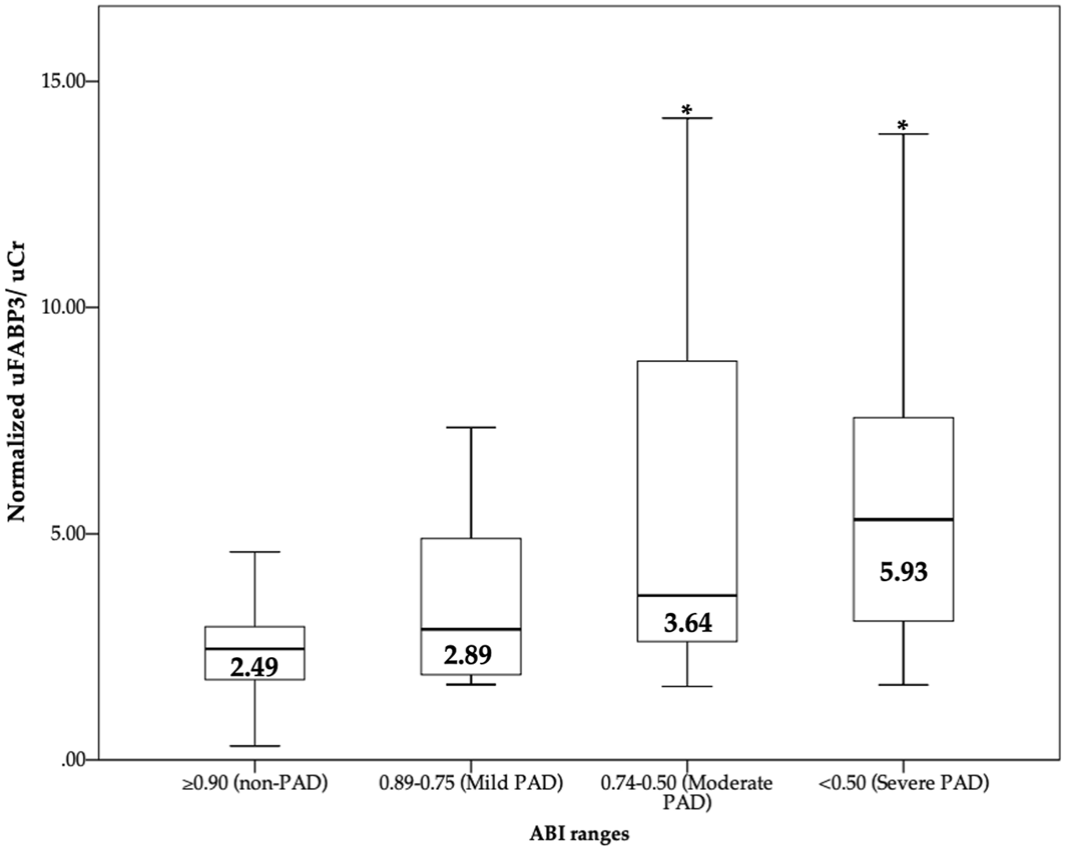
Boxplot illustrating the levels of normalized uFABP3/uCr among non-PAD controls (n = 65) and PAD patients stratified based on their ABI values (mild PAD: 0.89–0.75, n = 11; moderate PAD: 0.74–0.50, n = 29; severe PAD: < 0.50, n = 25). Significant difference in median uFABP3/uCr levels was noted between the moderate and severe PAD subgroups compared to non-PAD group, (p-value < 0.001). Values displayed in figure represents median uFABP3/uCr levels values in each subgroup. * p-value < 0.05 compared to non-PAD group.

To understand the association between uFABP3/uCr and the measured cardiovascular risk factors, subgroup analysis were conducted. Our analysis demonstrated that risk factors had no significant effect on uFABP3/uCr in both PAD and non-PAD patients (Table 2). We also investigated the association between uFABP3/uCr and PAD severity (based on the ABI) using spearman test. Our test demonstrated a significant negative correlation between uFABP3/uCr and ABI (ρ = − 0.436; p-value = 0.001; supplemental Fig. 1). Next, we investigated levels of uFABP3/uCr after stratifying PAD patients based on their ABI as per the ESVM guidelines^21^. The stratified groups were: mild PAD (ABI 0.75–0.9, n = 11), moderate PAD (ABI 0.74–0.50, n = 29) and severe PAD (ABI < 0.50, n = 25) . Our data shows a significant difference in median uFABP3/uCr levels between the moderate and severe PAD subgroups when compared to the non-PAD group (p-value < 0.001, for both) (Fig. 1). Although we noted a trend in increasing levels of uFABP3/uCr as the ABI worsens, no statistical difference was observed when comparing PAD subgroups specifically (p-value = 0.231).

**Table 2:** Median values of uFABP3/uCr in PAD and non-PAD patients with and without various PAD risk factors and comorbidities.

Condition	Non-PAD^¶^			PAD^¶^		
	Condition absent	Condition present	p-value^†^	Condition absent	Condition present	p-value^‡^
Age ≥ 65	2.44 (1.76–3.33)	2.60 (2.33–2.93)	0.820	3.69 (3.11–8.64)	4.77 (2.62–8.08)	0.915
Sex-male	2.46 (1.82–3.16)	2.49 (1.72–3.17)	0.989	5.70 (3.43–8.94)	3.63 (2.50–7.89)	0.159
Hypertension	2.24 (1.64–2.83)	2.70 (2.28–3.67)	0.067	3.90 (2.80–6.77)	4.45 (2.76–8.78)	0.383
Hypercholesteremia	2.45 (1.78–3.30)	2.53 (1.78–2.95)	0.837	5.81 (3.36–7.31)	3.90 (2.62–8.13)	0.437
Diabetes	2.45 (1.77–3.21)	2.65 (2.14–3.43)	0.513	3.81 (2.85–6.85)	5.87 (2.74–8.13)	0.609
Smoking	2.43 (1.72–3.66)	2.50 (1.80–3.04)	0.893	4.77 (3.13–9.08)	4.07 (2.76–8.08)	0.391
History of CAD	2.49 (1.77–3.23)	2.45 (1.80–3.04)	0.899	3.90 (2.90–8.54)	4.28 (2.48–7.32)	0.571

^¶^Median uFABP3/uCr, μg/g (interquartile range) for each subgroup.

^†^Difference among non-PAD patients.

^‡^Difference among PAD patients.

### Influence of confounding factors on uFABP3/uCr levels in patients with PAD

To investigate the independent association between uFABP3/uCr levels and PAD, multiple stepwise regression analysis was conducted. Our analysis demonstrated that uFABP3/uCr levels were strongly associated with PAD, even after adjusting for age and sex (model 1; OR, 2.02, 95% confidence interval (95% CI), 1.41–2.90, p-value = 0.001). Table 3 displays the results of several other regression models that demonstrate a significant association between uFABP3/uCr levels and PAD after adjusting for age, sex, prior history of CAD, smoking, eGFR and hypercholesteremia.

**Table 3:** Influence of individual factors on the odds ratios for PAD per one unit increase in normalized urinary fatty acid-binding protein 3.

Regression models	Odds ratio (95% CI)^‡^	p-value
Unadjusted model	1.93 (1.45–2.56)	0.001
Model 1 (adjusted for age and sex)	2.02 (1.41–2.90)	0.001
Model 1 + CAD	2.03 (1.38–2.97)	0.001
Model 1 + CAD + smoking	2.11 (1.40–3.19)	0.001
Model 1 + CAD + smoking + hypercholesteremia	2.15 (1.39–3.34)	0.001
Model 1 + CAD + smoking + hypercholesteremia + eGFR	2.34 (1.47–3.75)	0.001

*CAD* coronary arterial disease, *eGFR* estimated glomerular filtration rate, *CI* confidence interval.

^‡^Binary logistic regression models for PAD per one unit increase in uFABP3/uCr.

### Diagnostic potential of uFABP3/uCr for PAD

**Figure 2:**
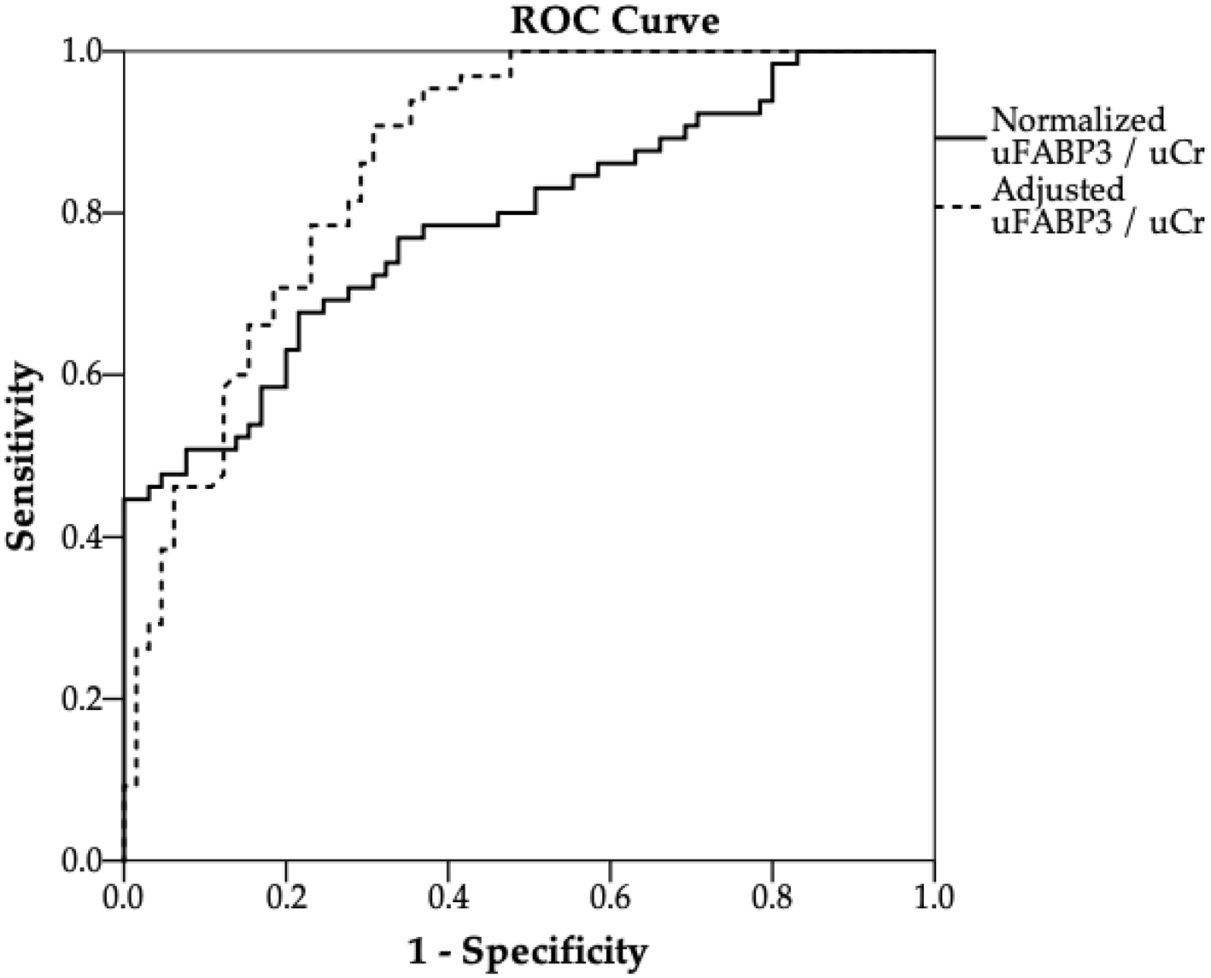
Receiver operating characteristic (ROC) curves of uFABP3/uCr for distinguishing patients with peripheral arterial disease (PAD, n = 65) from patients without PAD (non-PAD, n = 65) in an unadjusted model (solid line) and an adjusted model (dashed line). The area under the curve (AUC) and the 95% confidence intervals for uFABP3/uCr improved from 0.79 (95%: 0.71–0.87) to 0.86 (95%: 0.80–0.92) after adjusting for eGFR, age, hypercholesteremia, smoking and diabetes.

Lastly, a ROC analysis was performed to measure the diagnostic accuracy of uFABP3/uCr in diagnosing patients with PAD. Prior to adjusting for confounding factors, the AUC for uFABP3/uCr was 0.79 (95%: 0.71–0.87). After adjusting for age, eGFR, hypercholesteremia, smoking and diabetes, the ROC analysis for uFABP3/uCr demonstrated an improved AUC of 0.86 (95%: 0.80–0.92) (Fig. 2).

## Discussion

In this study, an association was demonstrated between PAD and uFABP3/uCr. Our data shows uFABP3/uCr is elevated in patients with PAD when compared to non-PAD controls, even after adjusting for potential confounding factors. Furthermore, the ROC analysis suggests potential for uFABP3/uCr in distinguishing PAD patients from non-PAD patients. Collectively, this data serves as a strong foundation for future studies investigating urine FABP3 as a biomarker for PAD.

Normally, biomarkers are used along with a clinical exam and radiographical investigations to enhance disease diagnosis. For instance, a diagnosis of acute myocardial infarction is reached after using a combination of several biomarkers (creatine kinase and troponin) in addition to an electrocardiogram and clinical symptoms. With regards to PAD, our research group previously identified that pFABP3 is potentially associated with PAD^12^, and through this study, have been able to demonstrate that uFABP3/uCr can serve as a potential diagnostic biomarker for PAD as well.

Fatty acid binding proteins (FABPs) play an important role in the trafficking of intracellular fatty acids^13,22^. Several studies have demonstrated the utility of uFABPs as potential biomarkers for diseased states. For instance, urinary FABP1 levels were noted to be higher in patients with septic shock when compared to patients with severe sepsis but without shock^23^. Tanaka et al. demonstrated that uFABP3 is elevated in patients with acute myocardial infraction^24^. Similarly, Nayashida et al. studied the influence of renal function on uFABP3 levels in patients undergoing a primary coronary artery bypass, and suggested, alongside others, that uFABP3 may be an early and sensitive biochemical marker for the diagnosis of myocardial injury in patients undergoing cardiac surgery^15,25^. Studies have also suggested that after myocardial injury, FABP3 filtration by the kidney can be impaired when creatinine clearance is decreased, resulting in lower levels of uFABP3^25,26^. Thus, uFABP3 may be utilized as a potential diagnostic biomarker for PAD in the absence of reduced creatinine clearance. Urinary FABPs may also serve as potential biomarkers for the earlier diagnosis of acute renal failure^27,28^.

The present study is not without limitations. First, there was a relatively small sample size. Second, due to the small sample size, we were not able to adjust for all potential confounding factors. Thirdly, the cross-section study design of this pilot trial did not allow for patient follow-up. Lastly, patients with acute myocardial infarction and renal failure were not included due to their confounding effect on uFABP3.

## Conclusions

In summary, our study revealed for the first time, that uFABP3/uCr is significantly elevated in patients with PAD. Our data demonstrated an independent association between uFABP3 and PAD, and ROC analysis demonstrated strong ability to discriminate, raising its potential as a tool for PAD diagnosis. Therefore, uFABP3 is a potential diagnostic biomarker for PAD; however, larger clinical trials are needed to confirm these findings.

## Supplementary Information

Supplementary Figure S1.
