# Supplementary material for: Urinary fatty acid binding protein 3 (uFABP3) is a potential biomarker for peripheral arterial disease
Source: Sci Rep. 2021 May 26;11:11061. doi: 10.1038/s41598-021-90395-0 (PMC8155078; doi:10.1038/s41598-021-90395-0)
Supplement: Supplementary file 1 — Supplementary Figure S1. [file 41598_2021_90395_MOESM1_ESM.docx]

Supplemental Figure 1:


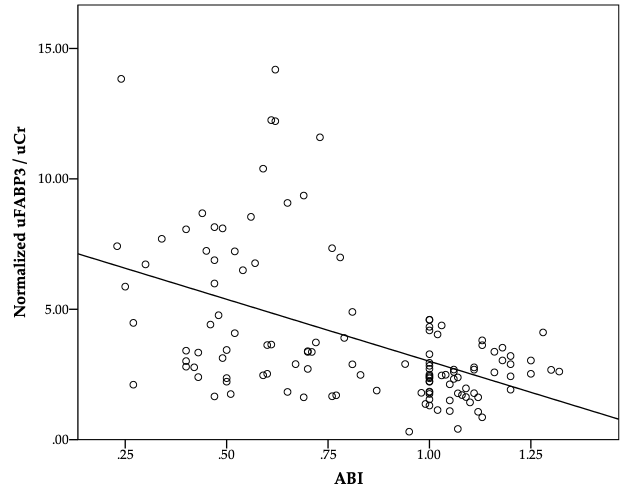


Supplemental Figure 1: Scatterplot representing the correlation between ABI and normalized uFABP3/uCr (μg/g) among all recruited patients (n=130). This figure demonstrates a significant inverse correlation between ABI and normalized uFABP3/uCr (μg/g) with a coefficient value of ρ= -0.436; p-value = 0.001.
